# Evaluation of the knowledge in seismotherapy of the nurses of the neuropsychiatry pole

**DOI:** 10.1192/j.eurpsy.2022.1931

**Published:** 2022-09-01

**Authors:** H. El Kefi, W. Kabtni, S. Borgi, I. Gafsi, C. Bencheikh, A. Oumaya

**Affiliations:** Hmpit, Psychiatry, Tunis, Tunisia

**Keywords:** general hospital, Nurses, seismotherapy, knowledge evaluation

## Abstract

**Introduction:**

Electroconvulsive therapy, also known as seismotherapy or electroshock therapy, consists of passing an alternating current of variable intensity between two electrodes placed on either side of the patient’s skull to create a generalized convulsive seizure with therapeutic effects.

**Objectives:**

To evaluate the knowledge of the nurses of the neuropsychiatry pole of the Military Hospital of Tunis in seismotherapy.

**Methods:**

Descriptive study, carried out in February 2021 in the pole of neuropsychiatry of the Military Hospital of Tunis (Services of psychiatry, neurology and neurosurgery). We developed for this study a form gathering sociodemographic questions and technical questions on seismotherapy (indications, contraindication, monitoring parameters …).

**Results:**

Thirty-nine (39) nurses agreed to answer the questionnaire. The average age was 37 years, 12 men and 27 women, with a sex ratio of 0.44. The majority (62%) of the participants had no idea about seismotherapy, 92% had never attended a session, 90% had no specific training, 87% thought that seismotherapy was indicated for all psychiatric illnesses. Seismotherapy was feasible on an empty stomach for 13% and after free and informed consent of the patient for only 33%.

**Conclusions:**

Although included in the nursing curriculum, the knowledge in seismotherapy of the nurses of the neuropsychiatry pole seems limited. A specific training program is indicated in anticipation of the establishment of a seismotherapy unit at the Military Hospital of Tunis.

**Disclosure:**

No significant relationships.

